# Intraoperative MRI Utilization by Moving Patients to the Magnet: Results From a Prospective Series of Brain Tumor Operations

**DOI:** 10.7759/cureus.77908

**Published:** 2025-01-24

**Authors:** Starlie C Belnap, Vitaly Siomin, John Candela, Sovietsky J Moreta-Paredes, Kevin Abrams, Michael McDermott

**Affiliations:** 1 Research, Miami Neuroscience Institute, Baptist Health South Florida, Miami, USA; 2 Neurological Surgery, Miami Neuroscience Institute, Baptist Health South Florida, Miami, USA; 3 Anesthesiology, Baptist Hospital of Miami, Miami, USA; 4 Neuroradiology, Miami Neuroscience Institute, Baptist Health South Florida, Miami, USA

**Keywords:** brain tumor, image-guided neurosurgery, intra-operative mri, neuro-imaging, post-operative imaging

## Abstract

Background and objectives

Intraoperative magnetic resonance imaging (iMRI) is valuable for assessing the extent of brain tumor resections and preventing repeat procedures for unexpected residual tumors. However, prolonged procedure times and restrictions on ferromagnetic materials deter widespread iMRI use. This study explored two methods to increase iMRI utilization, both involving transferring patients to the magnet after wound closure rather than moving the magnet into the operating room (OR).

Methods

A process improvement database of 40 consecutive patients undergoing iMRI for brain tumor surgery was analyzed. Phase-1, conducted between November 2020 and February 2021, involved transporting patients to the magnet and returning them to the OR for extubation. Phase-2, conducted between August 2021 and November 2021, designated an extubation area in the recovery room, eliminating the need to return patients to the OR. Diagnosis was used to match Phase-1 and Phase-2 cohorts with a retrospective comparative cohort (Phase-0) collected between June 2019 and August 2019. Several time intervals were recorded for analysis.

Results

A total of 57 cases were analyzed; 56% of patients were male, with a mean age of 56 years. iMRI volumes significantly increased in Phase-1 (100%, n = 20) and Phase-2 (100%, n = 19) compared to Phase-0 (22%, n = 4, p < 0.001). OR occupancy (p = 0.18) and anesthesia duration (p = 0.23) were statistically similar between groups, but the Phase-2 group demonstrated a clinically relevant median decrease of 78 minutes in case duration and a 41-minute reduction in anesthesia duration compared to Phase-0. Both Phase-1 and Phase-2 showed a significant 11-hour decrease in post-surgical MRI acquisition times compared to Phase-0 (p < 0.001).

Conclusion

Transferring patients to the magnet significantly increased iMRI utilization and facilitated immediate postoperative imaging without negatively impacting OR efficiency or anesthetic safety. Establishing an extubation site in the recovery room saved valuable OR time and reduced patient intubation time. The practice of “moving the patient, not the magnet,” combined with recovery room extubation, is now routine in our neurosurgery service.

## Introduction

A mobile intraoperative magnetic resonance imaging (iMRI) system was first developed at the University of Calgary at the Foothills Hospital for the postoperative evaluation of neurosurgical patients [1]. Many of these units were installed globally, and several other systems were developed concurrently [2]. One available system, the IMRIS system, employed a 1.5T conventional magnet suspended on a track, allowing the patient to remain in position while the magnet moved over the patient’s head for imaging [1]. One concern with moving the magnet was the potential movement of ferromagnetic surgical instruments [3,4], supplies, and anesthesia equipment [5] when the magnet was brought into the operating room (OR). Restrictions on the use and placement of ferromagnetic materials in the OR were dictated by the known strength of the static magnetic field and its ability to move objects [1-5].

As experience with intraoperative imaging grew, it became evident that certain tumor types (skull base tumors, pituitary adenomas, and low-grade gliomas) and their locations highlighted the importance of intraoperative imaging in ensuring the expected extent of resection [6-8]. Unexpected findings of residual tumor on imaging allowed the surgery to continue immediately, achieving the planned extent of resection without requiring a second OR visit. However, prolonged procedure times associated with moving the magnet into the OR became a deterrent for some neurosurgeons and departments [6,7]. This was the case at Baptist Hospital of Miami, where only 1-2 tumor patients were imaged monthly. To increase iMRI utilization, reduce postoperative imaging requests, minimize critical care transport needs, and decrease the number of return-to-OR cases, a task force was created to explore potential solutions. This initiative was led by the chief of neurosurgery and included OR leadership, imaging leadership, the anesthesiology medical program director, and the hospital chief of operations.

In this study, we report on the efforts of this task force. The aims were to increase iMRI utilization without prolonging procedure times or compromising patient safety, decrease postoperative imaging requests, and reduce the number of return-to-OR cases. Here, we outline the steps taken by the task force and the outcomes achieved in each phase.

## Materials and methods

A process improvement database was created to review consecutive patients undergoing tumor operations during Phase-1 and Phase-2. An image guidance technician (JC) was physically present to collect time intervals for both phases. All cases adhered to our standard of care institutional policy for intracranial tumors, which included obtaining patient surgical consent, iMRI scheduling, presurgical timeouts, and postoperative debriefs. This process ensures that patients are aware they will undergo imaging while asleep at the end of the procedure and that anesthesia and imaging staff are also informed. No changes were made to this process. Phase-1 data were collected between November 2020 and February 2021, and Phase-2 data were collected between August 2021 and November 2021. Case number assignments were used to prevent capturing personal health information or patient identifiers. The study was approved by the Baptist Health South Florida Institutional Review Board with full HIPAA compliance and a consent waiver of exemption (BHSF1759940).

**Figure 1:**
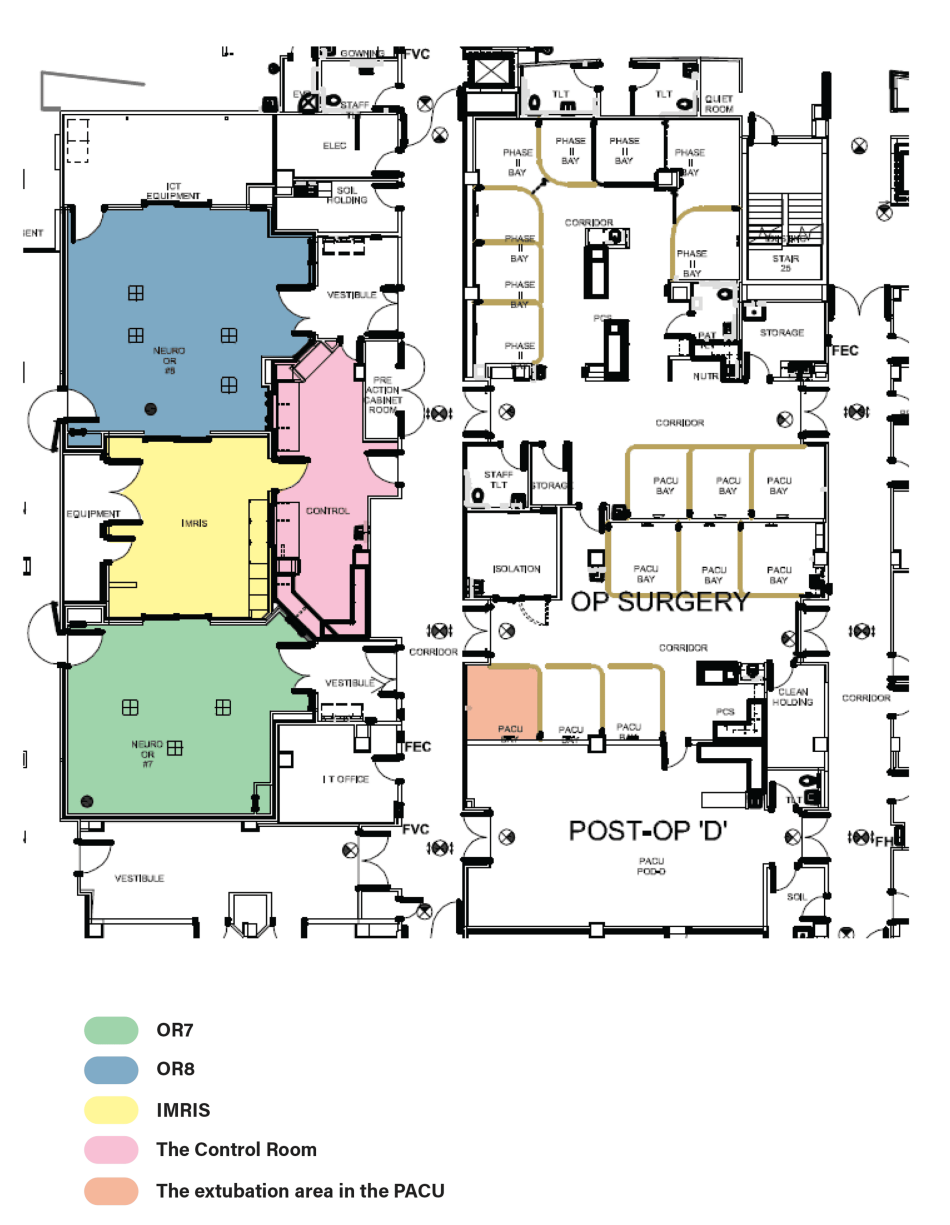
Operating Room Schematic Floor Plan Schematic floor plan for intraoperative MR imaging at our institution set up for two OR-room access. The iMRI (yellow) is situated between OR7 (green) and OR8 (blue) and joined together by the control room (pink).  The iMRI “home” position is located in yellow. The control room provides visibility to the iMRI home, OR7, and OR8.  The extubation suite was set up in the closest PACU room illustrated in orange to the control room.

**Figure 2:**
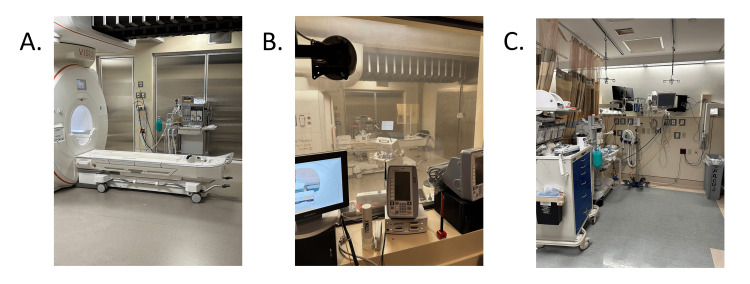
Intraoperative Imaging and Recovery Extubation Room Photographs (A) Photo of the iMRI with MR-compatible table in the home position. The MR-compatible table is used to move the patient from either room OR7 (75 feet) or room OR8 (69 feet) through the control room for imaging. (B) View from the control room to the intraoperative magnet in the home position illustrating imaging and general anesthesia monitoring equipment. (C) The recovery room extubation site with anesthesia equipment identical to that in the operating room. The distance from the control room to the recovery room bay is 65 feet.

The iMRI system we used was originally designed to move the magnet from its home position along ceiling rails into one of two adjacent operating rooms (OR7 and OR8), as illustrated in the schematic in Figure 1. Patients could also be transported to the iMRI on an MR-compatible table through the control room and scanned in the home position when the magnet was not deployed in the adjacent ORs. To increase the utilization of the magnet, we transported patients on the MR-compatible table to the magnet located in the home room (the central position between OR7 and OR8) after surgical closure while the patients were still under anesthesia, rather than deploying the magnet into the OR space (see Figure 2A). This allowed for the safe and sterile transport of patients to the home room. The control room was used in the standard manner to control imaging sequence acquisition and monitor anesthesia, as illustrated in Figure 2B. Following image acquisition, patients were returned to the OR for extubation and then transferred to the post-anesthesia care unit (PACU) for recovery. This initial strategy represents Phase-1 of the study, which focused on increasing iMRI volume and exploring its effects on OR occupancy.

The time intervals captured during Phase-1 included OR occupancy, anesthesia duration, total imaging time, patient arrival to intubation, procedure completion to imaging acquisition, procedure completion to extubation, and iMRI completion to extubation. OR occupancy was defined as the time from the patient entering the operating room to entering the PACU. Anesthesia duration was measured from the time of intubation to the time of extubation. Procedure completion to imaging acquisition was defined as the time from surgical closure to the start of the first post-surgical MRI image. This measure may include images obtained using a traditional stationary MRI within the radiology department rather than the iMRI.

In Phase-2, an extubation area was added to the recovery room to reduce the need to return to the OR once imaging was completed (see Figure 1). This area contained identical anesthesia monitoring equipment, as illustrated in Figure 2C. All the same time intervals were captured in Phase-2, with the addition of transportation time measured from imaging completion to entry into the extubation room. Phase-2 focused on maximizing OR efficiencies.

For comparative purposes, a cohort of consecutive surgical cases from June 2019 to August 2019 was retrospectively reviewed and matched by diagnosis to Phase-1 and Phase-2. This comparative cohort was labeled Phase-0. Time intervals for Phase-0 were abstracted from the medical record using operative time logs, anesthesia notes, and imaging timestamps. Cases with discrepancies in time notation were excluded from the analyses.

Other variables collected included subject age and sex, iMRI utilization volume, hospital length of stay (LOS), and discharge disposition. All collected variables were compared to Phase-0. Analysis of variance testing was used for normally distributed continuous variables, with post-hoc Tukey corrections for multiple comparisons, and nonparametric Kruskal-Wallis testing for nonnormal continuous variables. Means and standard deviations were provided where appropriate. All time intervals were reported using median hours:minutes with ranges. Categorical variables were evaluated using Pearson’s Chi-square and reported as percentages with sample sizes. All statistical analyses were performed using IBM SPSS Statistics for Windows, Version 27 (Released 2020; IBM Corp., Armonk, New York) at an alpha (α) level of 0.05.

## Results

A total of 44 patients were observed by the image guidance technician, with 24 cases during Phase-1 and 20 cases during Phase-2. Among these tumor cases, no unexpected hematomas were observed. No patients required re-intubation in the recovery room due to prolonged arousal or airway issues. Two patients, both with pituitary adenomas, were returned to the operating room for further resection and were excluded from the time analyses. Three additional subjects were excluded due to incomplete time records. The final sample size was 39 (Phase-1 = 20; Phase-2 = 19). For Phase-0, a total of 29 image-guided cases were reviewed and matched by primary diagnosis to cases observed during Phase-1 and Phase-2. Cases in Phase-0 were excluded for the following reasons: eight due to incomplete or contradictory time metrics in the medical records; one arrived intubated from the ICU; one returned to the OR for further resection; and one was an awake resection due to proximity to the motor cortex. The remaining 18 cases were retained for analyses. Primary diagnoses for each phase are detailed in Table 1.

**Table 1:** Patient Characteristics and Outcomes Means and standard deviations are shown for all continuous variables. Percentage and sample size are shown for all categorical variables. *Indicates statistical significance. LOS: length of stay; Home H/H: home with home health services; Rehab: inpatient rehabilitation services; SNF: skilled nursing facility; CT= computed tomography scan; Post-MRI= magnetic resonance imaging scan postsurgical transfer to the intensive care unit; iMRI=intraoperative MRI scan.

Variables	Phase-0, N = 18	Phase-1, N = 20	Phase-2, N = 19	Test Statistic	P-value
Age	55 (15.6)	57 (17.4)	55 (16.4)	F(2) = 0.11	0.89
Sex					
Male	56% (10)	35% (7)	53% (10)	χ^2^(2) = 1.92	0.38
Female	44% (8)	65% (13	47% (9)	χ^2^(2) = 1.92	0.38
Tumor diagnosis				χ^2^(10) = 7.35	0.69
Metastases	44% (8)	35% (7)	47% (9)	χ^2^(2) = 0.25	0.88
Glioma	28% (5)	35% (7)	16% (3)	χ^2^(2) = 2.71	0.26
Pituitary adenoma	11% (2)	10% (2)	16% (3)	χ^2^(2) = 1.6	0.45
Meningioma	11% (2)	10% (2)	21% (4)	χ^2^(2) = 1.0	0.61
Skull base	6% (1)	10% (2)	0% (0)	χ^2^(2) = 1.0	0.61
LOS	11 (7.2)	9.5 (10)	10 (9.7)	F(2) = 1.57	0.43
Discharge disposition				χ^2^(8) = 5.5	0.70
Home	56% (10)	55% (11)	63% (12)	χ^2^(2) = 0.18	0.91
Home H/H	22% (4)	20% (4)	21% (4)	χ^2^(2) = 0.0	1.0
Rehab	17% (3)	25% (5)	11% (2)	χ^2^(2) = 1.4	.5
SNF	5% (1)	0% (0)	0% (0)	N/A	N/A
Expired	0% (0)	0% (0)	5% (1)	N/A	N/A
Imaging volume				χ^2^(2) = 40.21	0.01
CT	11% (2)	0	0	N/A	N/A
Post-MRI	67% (12)	0	0	N/A	N/A
iMRI	22% (4)	100% (20)*	100% (20)*	χ^2^(2) = 11.21	0.01

For the 57 patients included in the analyses, the average age was 56 (SD = 16), with 56% listed as male. No statistical differences in age (F(2) = 0.12, p = 0.89), sex (χ²(2) = 1.92, p = 0.38), or diagnosis (χ²(10) = 7.34, p = 0.69) were observed between any phases. Additionally, no statistical differences were observed in hospital length of stay (F(2) = 0.87, p = 0.42) or discharge disposition (χ²(8) = 5.5, p = 0.70) (Table 1). However, significant differences were observed for iMRI case volumes (χ²(2) = 40.21, p < 0.001). In Phase-0, iMRI was only utilized in 22% (n = 4) of cases compared to 100% utilization in Phase-1 and Phase-2. The most common imaging modality used in Phase-0 was traditional MRI (67%, N = 12). The least common imaging modality in Phase-0 was computed tomography (11%, N = 2). For all 12 cases where traditional MRI was used, the patients were transported from the intensive care unit to the radiology department. For the four patients where iMRI was utilized, post-procedure imaging was captured within the OR under sedation, where the magnet was deployed to the patient. 

When evaluating the time intervals (Table 2), patient arrival to intubation, anesthesia duration, and total imaging time did not statistically vary by phase. While statistical benefit was not observed for anesthesia duration, there was a notable clinical benefit of a median 43-minute decrease between Phase-0 and Phase-2 and a 93-minute decrease between Phase-1 and Phase-2. Kruskal-Wallis analysis indicated a statistically significant increase in procedure completion to extubation (H(2) = 34.98, p < 0.001). As anticipated, patients remained intubated longer in Phase-1 (M = 1:17, 0:25-2:00) and in Phase-2 (M = 1:40, 0:57-2:10) when compared to Phase-0 (M = 0:03, 0:02-1:13). Conversely, there was a statistically significant decrease from imaging completion to extubation (H(2) = 6.73, p < 0.05). Phase-0 patients’ median imaging completion to extubation times were 46 minutes (0:16-1:34), whereas for Phase-1 and Phase-2 they were 14 (0:04-0:31) and 15 (0:10-0:40) minutes, respectively. It is important to note that for Phase-2, the reported imaging completion to extubation included the median transportation time of 5 minutes (range = 0:01-0:17). We also observed a statistically significant decrease in post-surgical image acquisition (H(2) = 29.47, p < 0.001). For the 16 cases in Phase-0 that acquired an MRI either intraoperatively or post-procedure, the median acquisition time was 11:20 (range = 0:32-23:22), while for Phase-1 and Phase-2 cases, the median acquisition time was 15 minutes (range = 0:05-0:56) and 28 minutes (range = 0:02-0:54), respectively, reflecting an 11-hour decrease. When comparing iMRI Phase-0 cases only (M = 0:30, 0:21-0:36), Phase-1 (M = 0:15, 0:05-0:56) and Phase-2 (M = 0:28, 0:02-0:54) post-surgical MRI acquisition times remained statistically similar (H(2) = 4.68, p = 0.06). Lastly, total OR occupancy remained statistically similar between all phases (H(2) = 3.43, p = 0.18), with a promising 49-minute clinical OR efficiency improvement observed from Phase-0 to Phase-2 and a 1-hour improvement from Phase-1 to Phase-2.

**Table 2:** Surgical Time Intervals Medians and interquartile ranges are shown for all variables. ^~^Indicates clinical significance compared to Phase-0. ^a^Includes all postsurgical imaging techniques (traditional and iMRI). ^b^Indicates Phase-0 sample size restricted to iMRI cases only, n = 4. *Indicates Kruskal-Wallis statistical evaluation was significant, p < 0.05. ^+^Indicates post-hoc test results compared to Phase-0 were of statistical significance, p < 0.05.

Variables	Phase-0, N = 18	Phase-1, N = 20	Phase-2, N = 19	H-test	P-value
OR occupancy	6:15 (3:23)	6:51 (2:30)	4:57 (2:21)^~^	3.43	0.18
Anesthesia Dur	5:48 (2:47)	6:38 (2:13)	5:07 (2:27)^~^	2.96	0.35
Procedure to postimaging^a^	11:20 (15:38)	0:15 (0:18)+	0:28 (0:27)^+^	29.47	0.01*
Procedure to iMRI imaging^b^	0:30(0:12)	0:19 (0:18)+	0:28 (0:27)	6.57	0.04*
Procedure to extubation	0:03 (0:06)	1:17 (0:33)+	1:40 (0:31)^+^	34.99	0.01*
iMRI to extubation^b^	0:46 (1:05)	0:14 (0:14)+	0:15 (0:13)^+^	6.73	0.04*

## Discussion

Intraoperative imaging has advantages for determining the extent of resection immediately after surgery while the patient is still under general anesthesia. Unexpected findings allow the surgeon to make quick decisions about the need to return to the operating room for additional resection, and this may be more common with pituitary adenomas, low-grade gliomas, and skull base tumors [6-9]. In our study, iMRI potentially prevented two "next day" return-to-OR cases observed in the comparative sample. For both of these cases, the need for additional resection was identified early while the patient was still under anesthesia, allowing the surgeon to immediately return to the OR to complete the case. This result is similar to that reported by Jankovski et al., where iMRI imaging prevented three next-day return-to-OR cases.

One of the main deterrents to iMRI utilization, by moving the magnet on rails into the operating room, is prolonged OR duration [7,10]. In our study, we successfully demonstrated that consistent postoperative iMRI acquisition can be done without prolonging procedure times when the patient is moved to the magnet and a designated extubation suite is employed. Neither the Phase-1 nor Phase-2 cohorts statistically differed in OR occupancy when compared to Phase-0. Our results indicated that the addition of the extubation suite reduced OR occupancy by a median of 49 minutes compared to the pre-COVID historical dataset. While the observed time savings were not consistent enough to reach statistical significance, this time reduction is clinically meaningful in improving OR efficiency and may demonstrate long-term cost savings [11]. A larger multicenter study will be needed to explore this possibility.

Efficiencies in postoperative imaging can reduce imaging requests and patient transport out of the critical care setting, thereby reducing the risks associated with intrahospital transport [12]. In the current study, we were able to decrease image acquisition time by approximately 11 hours and reduce the imaging request burden by 12 cases. Additionally, our study demonstrated that transporting the patient to the intraoperative magnet did not delay image acquisition when compared to traditional iMRI cases. Therefore, iMRI reduced the demand on the neuroradiology department and provided patients with the additional comfort of being imaged under general anesthesia.

Another deterrent frequently identified is the use of ferromagnetic materials in the OR. With the ever-improving imaging quality of stronger magnets (1.5T, 3T, 7T), there is an equally advancing concern about the impact of magnetic fields on surgical tools, anesthesia equipment, and even air change filtration systems needed to maintain sterility within the OR [4]. The practice of "moving the patient to the magnet" helps alleviate some of these concerns because the operating space is shielded from the magnet docked in an adjoining room, greatly decreasing the impact of the magnetic field. Furthermore, the control room allows anesthesiology to safely monitor the patient (Figure 2B), reducing concerns about moving anesthesia equipment between rooms. Interestingly, the addition of the extubation suite showed a 43-minute clinical reduction in total anesthesia duration when compared to the pre-COVID historical dataset. This decrease in anesthesia duration directly improves patient safety and may translate to more cost benefits.

This study was a single-center prospective consecutive sample study design, which limits the generalizability of the results to other institutions. Despite this limitation, the study successfully demonstrated that it is possible to improve iMRI utilization at a single center by moving patients to the magnet and using an extubation suite. Another potential concern is the difference in time interval capture between the phases. Time intervals for Phase-1 and Phase-2 ("moving the patient to the magnet") were captured through direct observation by a designated technician, whereas data for the comparison cohort in Phase-0 ("moving the magnet to the patient") were abstracted solely from the medical record. These differences may have introduced bias and unintentionally skewed the results. It is common for various time-capturing systems to be miscalibrated or slightly out of sync. This mismatch may have resulted in cases being erroneously excluded from the comparison group and potentially altering the results in the observation groups. Nevertheless, even with our conservative approach of excluding cases with mismatching times, we demonstrated increased utilization and an overall reduction in post-surgical imaging acquisition. Future multi-center studies should account for these limitations to improve the reliability and validity of the results.

While we recognize the limited generalizability of our results, we are confident that centers with a similar floor plan may potentially increase their iMRI utilization by adopting the practice of moving patients to the magnet to garner immediate post-operative imaging. We further encourage similar institutions to consider adding an extubation area to the recovery room to improve OR occupancy durations.

## Conclusions

In summary, "moving patients to the magnet" successfully increased the utilization of iMRI at our institution and provided immediate access to postoperative imaging for assessment without negatively impacting OR efficiency or anesthetic safety. The creation of the extubation suite saved valuable operating room time and reduced patient intubation time. Because of these observed benefits, iMRI acquisition with an extubation area in the recovery room is now routine in our neurosurgery service.
